# Organic Food: A Comparative Study of the Effect of Tomato Cultivars and Cultivation Conditions on the Physico-Chemical Properties

**DOI:** 10.3390/foods4030263

**Published:** 2015-07-10

**Authors:** Jacqueline C. Araujo, Samuel F. P. Telhado

**Affiliations:** Embrapa Mandioca e Fruticultura, Cruz das Almas, Bahia 44380-000, Brazil; E-Mail: samuel.telhado@embrapa.br

**Keywords:** *Solanum lycorpersicum* L., organic production system, organoleptic traits, physico-chemical characteristics

## Abstract

The objective of this review was to present an update of the currently managed studies on the characterization physical, chemical, and sensory analysis of several tomato cultivars. This review has indicated the importance of farming system and genotype on sensory and biochemical characteristics. It is necessary to use selected genotypes responding positively to organic farming in terms of sensory, biochemical characteristics and productivity aspects and to evaluate systems over more than one year of sampling.

## 1. Introduction

The tomato crop (*Solanum lycopersicum* L.) has a great importance in the world, and its production, in 2013, reached 163.9 million tons and average yield of 34.7 tons per hectare [1]. The tomato is classified as a functional food, for having good levels of vitamins, minerals, and especially lycopene, a carotenoid pigment that provides red color and has antioxidant qualities [2].

The quality of the tomato is affected by genetic foundation, developing conditions, used inputs, and aging during postharvest storage.

The quality of a vegetable can be characterized by features such as appearance, texture, safety, flavor and nutritional value. The appearance is the main characteristic because it defines the product commercialization value [3].

The growing management of tomatoes, however, is highly influenced by pulverization of pesticides, so, there is the requirement for improving tomato production, and give consumers superior flavor and quality to reach their expectations [4].

To produce organic food, it is necessary to use inputs and methods that improve the ecological equilibrium of natural systems. This happens because organic vegetable is grown without pesticides, herbicides, highly soluble fertilizers and genetically modified organisms. The value of the organic product is not only in the product itself, but also in its production process.

Thus, personal principles are one of the important reasons found to encourage the purchase of organic food [5], as well as health concerns. So, in addition to the agricultural aspects, consumers also consider the environmental and social factors. These characteristics of quality from organic products classify it as a “belief”, seeing that they exhibit highly specified attributes of quality, non-identifiable by simple observation, neither after the purchase [6].

Several researches show that the demand of organic agriculture has increased, because this kind of product is identified by the consumers as a healthy product. The majority of the consumers of organic products, usually are women between 30 and 50 years; with high instruction level; belonging to the middle class and with purchase behavior diversified. The main motivation for purchase is the familiar health, followed by the absence of pesticide use, the biological value, the taste, the aroma and the environmental concern [6].

The total of organic land in the world has reached 43.1 million hectares in 2013, with 2 million organic producers. The total area under organic vegetable production has tripled, from 105 thousand hectares in 2004, to 305 thousand hectares in 2013. However, it represents only 0.5% of the total area of vegetables grown in the world. The United States, China, Mexico and Italy are the countries with the largest organic vegetable areas, all of them with more than 20 thousand hectares each [7]. Despite the high growth, the organic farming comprises a small area of the total acreage in the world, probably due to the need for more information about the agronomic development of crops under organic management, justifying the importance of a larger effort in research for new agricultural practices.

Many approaches, including sensory, physical and chemical are currently in use to determine tomato quality. This review reveals the importance of tomato cultivars on their quality.

## 2. Material and Methods

An extensive bibliographical search was conducted using the following keywords: organic tomato; physicochemical characteristics tomato; sensory characteristics tomato and organic farming tomato. The articles that covered physicochemical and sensory characteristics were analyzed and the summary of the higher significant differences between conventional and organic tomato fruit can be found in Table 1.

## 3. Literature Review

### 3.1. Physicochemical Characteristics

The quality of the tomato for fresh market, in organic or conventional production system, is determined by appearance, firmness and flavor; although the quality of the processed tomato is determined essentially by soluble solids, color, pH and firmness [8].

Several studies were performed in order to evaluate the physicochemical and sensory characteristics of tomato fruits [9,10,11], and its yield [12], under organic management.

Other researchers have compared qualitative parameters in fruits and vegetables produced under organic and conventional production system; however, there is no conclusive evidence on the nutritional or qualitative superiority of either system [13].

After analyzing data from 10 years regarding the influence of different crop management, organic and conventional, the medium levels of flavonoids quercetin and kaempferol, in organic tomatoes were 79% and 97% higher than those in conventional tomatoes, respectively [14].

The physico-chemical and sensory parameters of 14 experimental and commercial fresh tomato cultivars under the organic production system were analyzed and all the cultivars presented reasonable quality of the fruits, with SS/TA ratio values higher than 16 and SS content higher than 4 ºBrix [11].

Different variables influenced the nutritional quality of the organic product. The type of fertilizer used influenced the concentration of antioxidant components in tomatoes, increasing the amount of phenolic compound and ascorbic acid in tomatoes, which were fertilized with organic fertilizer [15].

To assume that the means of production is the only thing responsible for the differences is a risk, because there are a lot of variables that affect the quality and the nutrition of fruits and vegetables, such as: cultivar, climate, soil type, irrigation practices, fertilization, maturity at harvest and post-harvest care, which also affect the quality of the harvested products, and to determine the level of importance within the production system, these variables should be controlled [13].

In recent research, organic tomato showed higher quality when compared with the ones produced in the conventional system, based on the level of soluble solids ºBrix) and Bostwick values of consistency [16].

Other researchers showed a higher number of studies with favorable results to the organic system, mainly concerning to the lower level of nitrate when compared to the conventional system [17,18]. Another study compared the quality of tomato fruits with increasing doses of nitrogen (N) and with or without the use of organic manure, it found that there was no difference between the pH of the fruit, and the soluble solids content, with increasing nitrogen rates, the presence or absence of organic manure. However, the level of nitrate enlarged when happened the increasing of N rates without adding organic manure, whereas the level of nitrate remained constant with the addition of organic matter [19].

Tomatoes fruits were bought from the same cultivar (“Daniella”), in two consecutive years (2010 and 2011), at the same degree of maturity and the same size to study the differences in the phenolic content of organic and conventional tomatoes in the flavonoids (kaempferol, quercetin and rutin), flavonones (naringenin and naringenin-7-*O*-glucoside), flavones (apigenin-7-*O*-glucoside) and hydroxycinnamic acids (ferulic, p-coumaric, caffeic acid and chlorogenic). The phytochemical concentration, including the level of flavonoids, showed higher value in the fruits produced in the organic system [20]. Another study, concerning the content of phenolic compounds and ascorbic acid, had found higher levels in tomatoes from organic system when compared with conventional system [21]. This study corroborated another research that also found higher content of phenolic compounds in organic tomato in three consecutive years (2008, 2009 and 2010) in Hungary, with nine different cultivars. However, in the same work higher values for soluble solids, carbohydrate and lycopene were found in conventional tomatoes [22].

Studies reported that the fruits from organic farming have higher levels in all the compounds analyzed: ascorbic acid (+30%), lycopene (+20%), total phenolics (+24%), and flavonoids (+21%) [23]. It also supports another study demonstrating that tomatoes from organic farming accumulate higher levels of vitamin C (+55%) and phenolic compounds (+139%) than those from conventional farms. For the authors, the reason why organic tomatoes had higher levels of these compounds can be because the plants are more exposed to stress in organic farming [24].

A review compared studies that evaluated nutritional value, food safety and sensorial quality. According to the authors, the nitrate level was statistically lower in food produced under the organic system than in the conventional system; however, the nutrient content did not show any difference [25].

In a two-year research, the total sugar content, organic acids, vitamin C and phenolic compounds (quercetin-3-*O*-rutinoside, myricetin and quercetin) were evaluated. The organic tomatoes showed higher levels of these compounds, when compared with tomatoes derived from the conventional production system [26].

Organic ketchup when compared with conventional ketchup showed higher total carotenoids and lycopene levels [27]. On the other hand, studies [28,29] demonstrated that total lycopene content is not affected by organic farming. According to another work, the levels of vitamin C, total acidity, lycopene and carbohydrate, among the fruits of tomatoes from the organic and the conventional system, also did not have significant differences [30].

**Table 1:** Summary of the significant differences between conventional and organic tomato fruit.

Characteristics	Higher in Organic	Higher in Conventional	No Difference	References
Carotenoids	X			[27]
Lycopene	X			[23,27]
			X	[28,29,30]
		X		[22]
Ascorbic acid	X			[21,23]
Flavonoids	X			[20,23]
Carbohydrate			X	[30]
		X		[22]
Total sugar content	X			[26]
Organic acids	X			[26]
Vitamin C	X			[24,26]
			X	[30]
Total soluble solids	X			[13,16]
			X	[19]
		X		[22]
Total acidity			X	[13,30]
Higher Consistency (Bostwick values)	X			[13,16]
phenolic compounds (quercetin-3-*O*-rutinoside, myricetin, quercetin, kaempferol)	X			[14,21,22,23,24,26]
Level of nitrate		X		[17,18,25]

### 3.2. Sensory Properties

In recent research, 14 cultivars were analyzed by 75 individuals, using a hedonic scale of seven points, but only eight cultivars (IAC 4, IAC 6, Netuno, Bari, IAC 1, IAC 5, HTV 0601 and Debora Victory) obtained the best scores in all sensorial traits evaluated (flavor, color of the pericarp, internal color, texture and overall impression) [11].

Another work evaluated 10 accessions of organic cherry tomatoes by a trained panel using the Quantitative Descriptive Analysis (QDA), and similarly by 80 tomato consumers for the acceptance of appearance and intention to purchase. Subsequently, the results reached of the integration of data from the trained panel and consumers made a multidimensional map that elucidated of consumer liking of tomatoes in relation to the appearance, *i.e.*, leads of liking/disliking were identified. In addition, the results showed that tomatoes with a round shape and a red color (reddish) were the most liked cherry tomatoes [3].

In the literature, different authors revealed that growing methods had no statistically significant effect on sensory parameters, in particular on aroma [31], because these characteristics were influenced mainly by cultivars [11,28,32].

## 4. Conclusions

This review has indicated the importance of farming system and genotype on sensory and biochemical characteristics. It is necessary to use selected genotypes responding positively to organic farming in terms of sensory, biochemical characteristics and productivity aspects and to evaluate systems over more than one year of sampling.
